# Assessing white matter microstructural changes in idiopathic normal pressure hydrocephalus using voxel-based R2* relaxometry analysis

**DOI:** 10.3389/fneur.2023.1251230

**Published:** 2023-09-05

**Authors:** Yuya Kano, Yuto Uchida, Hirohito Kan, Keita Sakurai, Susumu Kobayashi, Kento Seko, Keisuke Mizutani, Toshihiko Usami, Koji Takada, Noriyuki Matsukawa

**Affiliations:** ^1^Department of Neurology, Toyokawa City Hospital, Toyokawa, Japan; ^2^Department of Neurology, Nagoya City University Graduate School of Medical Sciences, Nagoya, Japan; ^3^The Russell H. Morgan, Department of Radiology and Radiological Science, Johns Hopkins University School of Medicine, Baltimore, MD, United States; ^4^Department of Integrated Health Sciences, Nagoya University Graduate School of Medicine, Nagoya, Japan; ^5^Department of Radiology, National Center for Geriatrics and Gerontology, Ōbu, Japan; ^6^Department of Radiology, Toyokawa City Hospital, Toyokawa, Japan

**Keywords:** idiopathic normal-pressure hydrocephalus, magnetic resonance imaging, R2* relaxometry analysis, quantitative susceptibility mapping, voxel-based analysis

## Abstract

**Background:**

R2* relaxometry and quantitative susceptibility mapping can be combined to distinguish between microstructural changes and iron deposition in white matter. Here, we aimed to explore microstructural changes in the white matter associated with clinical presentations such as cognitive impairment in patients with idiopathic normal-pressure hydrocephalus (iNPH) using R2* relaxometry analysis in combination with quantitative susceptibility mapping.

**Methods:**

We evaluated 16 patients clinically diagnosed with possible or probable iNPH and 18 matched healthy controls (HC) who were chosen based on similarity in age and sex. R2* and quantitative susceptibility mapping were compared using voxel-wise and atlas-based one-way analysis of covariance (ANCOVA). Finally, partial correlation analyses were performed to assess the relationship between R2* and clinical presentations.

**Results:**

R2* was lower in some white matter regions, including the bilateral superior longitudinal fascicle and sagittal stratum, in the iNPH group compared to the HC group. The voxel-based quantitative susceptibility mapping results did not differ between the groups. The atlas-based group comparisons yielded negative mean susceptibility values in almost all brain regions, indicating no clear paramagnetic iron deposition in the white matter of any subject. R2* and cognitive performance scores between the left superior longitudinal fasciculus (SLF) and right sagittal stratum (SS) were positively correlated. In addition to that, R2* and gait disturbance scores between left SS were negatively correlated.

**Conclusion:**

Our analysis highlights the microstructural changes without iron deposition in the SLF and SS, and their association with cognitive impairment and gait disturbance in patients with iNPH.

## 1. Introduction

Normal-pressure hydrocephalus (NPH) is a clinical entity proposed by Adams et al. (1) that presents as a triad of cognitive impairments, gait disturbances, and urinary incontinence. This disease is characterized by enlarged ventricles; nonetheless, the cerebrospinal fluid (CSF) pressure is within the normal range, and symptoms improve following shunt surgery.

Magnetic resonance imaging (MRI) findings of idiopathic NPH (iNPH), also known as disproportionately enlarged subarachnoid space hydrocephalus (DESH), include ventriculomegaly, enlarged Sylvian fissures, tight high-convexity and medial subarachnoid spaces, and localized sulcal dilatation (2, 3). Furthermore, periventricular and deep white matter (WM) changes are observed more frequently in these patients than in healthy controls (HC) (4). Several studies in iNPH patients have reported that diffusion tensor imaging (DTI) can detect microstructural changes in the WM that are related to symptoms (5, 6). Studies vary in confirming the presence and location of changes in fractional anisotropy (FA) and mean diffusivity (MD), whereas WM alterations include an increase in FA and/or MD. These diffusion-based measures, however highly sensitive, have distinct limits in accurately identifying tissue microstructural conditions. This may be due to their varying susceptibility to factors such as axon density, cell swelling, fiber structure, and axon radius (7).

R2* relaxometry analysis can assess iron deposition on multiple spoiled gradient echo sequences (mGRE) and is sensitive to WM microstructural changes such as myelin loss (8, 9). This approach has advantages over DTI, such as a shorter imaging time, higher spatial resolution, and sensitivity to environmental changes in WM. According to research, slight demyelination, axonal and oligodendrocyte loss, and an increase in extracellular space indicate a significant decrease in R2* (10). On the other hand, quantitative susceptibility measurement (QSM) is highly sensitive to iron deposition (11). Therefore, R2* relaxometry analysis can be used in combination with QSM to differentiate between microstructural changes and iron deposition, presenting an estimation of the biological particularity of WM and promoting our understanding of pathological changes in WM in iNPH (10).

Herein, we report the combination of R2* relaxometry with QSM in patients with iNPH vs. HCs. We aimed to evaluate the microstructural changes in the WM of patients with iNPH using voxel- and atlas-based R2* and susceptibility analyses. Furthermore, we aimed to investigated the association between R2* and clinical presentations in iNPH. We hypothesized that WM microstructural changes without iron deposition are associated with clinical presentations in patients with iNPH.

## 2. Materials and methods

### 2.1. Participants

In this retrospective, single-center, observational study, we enrolled 16 patients with clinically diagnosed possible or probable iNPH (nine men and seven women; mean age ± SD:81.9 ± 3.4 years) and 18 age- and sex-matched healthy controls (HCs; 10 men and eight women, mean age ± SD:81.4 ± 3.8 years). All patients presented with DESH and were diagnosed according to the diagnostic criteria outlined in the Japanese guidelines for iNPH management (12). Cognitive functions were assessed using the Mini-Mental State Examination (MMSE) and activities of daily living were scored using the modified Rankin Scale (mRS). To classify the symptoms of patients with iNPH, we scored gait, cognitive function, and voluntary urinary control function using the INPH grading scale (INPHGS 0 = normal; 4 = severe disability) (13). This study was approved by the Toyokawa City Hospital Institutional Review Board.

### 2.2. Magnetic resonance imaging acquisition

Magnetic resonance imaging scans were acquired using a 3 T magnetic resonance imaging scanner (Ingenia 3.0 T; Philips Medical Systems International, Best, The Netherlands). A three-dimensional (3D) multiple spoiled gradient echo sequence (mSPGR) was acquired in the axial plane to estimate R2* and perform QSM. The mSPGR scan parameters were as follows: FOV, 192 × 192 × 144 mm^3^; acquisition matrix size, 192 × 192 × 144; TR, 34.9 ms; TE, 6.0–30.8 ms at 6.2-ms intervals; number of echoes, 5; parallel imaging factor, 2; and flip angle, 15^°^. Then, to spatially normalize R2* and the QSM values, the 3D T1-weighted images were acquired using a magnetization-prepared spoiled gradient echo sequence (MP-SPGR) in the sagittal plane and with the following scan parameters: FOV, 256 × 256 mm^2^; acquisition matrix size, 224 × 224; number of slices, 144; slice thickness, 1.2 mm; TR, 4.2 ms; TE, 2.3 ms; inversion time, 900 ms; interval between successive inversion pulses, 2,500 ms; parallel imaging factor, 2; and flip angle, 9°. T2-weighted turbo spin echo, fluid-attenuated inversion recovery, diffusion-weighted, and T2*-weighted images were acquired routinely to identify and exclude any brain abnormalities.

### 2.3. R2* relaxometry analysis for evaluation of microstructural changes

To detect microstructural WM changes, R2* relaxometry analysis was performed using multiple-magnitude images. The R2* value was fitted from the multi-magnitude data to the mono-exponential R2* decay using auto-regression on linear operations, which provides a fast and accurate R2* estimation using the maximum-likelihood fit of an autoregressive model (14). Note that excessive signal loss due to macroscopic field inhomogeneity was negligible in the WM. The macroscopic field inhomogeneity effect mainly occurs close to the brain-air interface, leading to signal leakage in neighboring voxels. However, signal loss due to signal leakage was minimized in the WM at the center of the brain in the TE range we used (10).

### 2.4. QSM analysis for evaluation of iron deposition

Because iron deposition as well as microstructural WM changes contribute to the R2* value, simultaneous evaluation is necessary to distinguish between the two (15). We thus performed the QSM analysis, which can detect iron deposition within a voxel, using the same dataset that was entered into the R2* relaxometry analysis. The phase images in the mSPGR were subjected to Laplacian-based phase unwrapping (16). Each unwrapped phase of each TE was then removed from the background field caused by the tissue-air interface using sophisticated harmonic artifact reduction for phase data with varying kernel sizes (17–19). Weighted averaging was performed on the local fields of each TE based on the estimated R2*map (17, 18). The susceptibility map was reconstructed from the local field map using improved sparse linear equations and the least-squares method (20, 21). The mean CSF susceptibility value in the lateral ventricles extracted from the R2* map was subtracted from the susceptibility map as a zero reference for the susceptibility value (22).

### 2.5. Preparation of voxel-based analyses

T1-weighted structural images were segmented into WM, gray matter, and CSF using statistical parametric mapping 12 (SPM12). We visually confirmed that there were no segmentation errors. The WM images were spatially normalized, and the volumetric information preserved using a study-specific template generated by diffeomorphic anatomical registration and the exponentiated Lie algebra algorithm (23). The mask for voxel-based analysis was created with the SPM12 toolbox (24). To transform the R2* and QSM maps into normalized space, the magnitude image of the first echo in the mSPGR was co-registered with the T1-weighted images (25–30). The R2* and QSM maps were then transformed into standard space using the same transformation parameter**s** (10). Both images were smoothed using an 8-mm Gaussian kernel.

### 2.6. Statistical analyses

Statistical analyses were performed using Stata 17.0 (StataCorp, College Station, TX, United States). The Mann–Whitney U-test for continuous data and Pearson’s chi-square test for categorical data were used to identify significant intergroup differences. A voxel-wise one-way analysis of covariance (ANCOVA) in SPM 12 was used to compare R2* and QSM through the whole brain between groups. Using the family-wise error (FWE) method and clusters of ≥100 contiguous voxels, a significance level of *p* = 0.05 was applied with multiple comparison corrections. The WM atlas created by Johns Hopkins University (JHU-WM atlas) was applied in the atlas-based ANCOVA to determine the anatomical locations that showed significance in the voxel-based analysis (31). Finally, the association between R2* and clinical presentations was examined using partial correlation analysis. All variables were adjusted for age and sex (used as covariates in the analyses).

## 3. Results

### 3.1. Participant characteristics

The participant demographics are summarized in Table 1. Participants were age- (*p* = 0.65) and sex-matched (*p* = 0.97). The iNPH and HC groups exhibited significant differences in MMSE (*p* = 0.02) and mRS (*p* < 0.001) scores. Patients with iNPH presented with gait disturbances (94%), cognitive dysfunction (100%), and urinary dysfunction (81%), with cognitive symptoms being the most severe.

**Table 1:** Demographic and clinical characteristics of participants.

	iNPH	Control	*p* value
*n* [male/female]	16 [9/7]	18 [10/8]	0.97
Age (years)	81.9 (3.4)	81.4 (3.8)	0.65
MMSE	18.3 (6.2)	23.3 (4.0)	0.02
mRS	2.6 (0.7)	1.1 (0.3)	< 0.001
iNPHGS			
Gait disturbance	1.8 (0.5)	-	
Cognitive impairment	2.4 (0.7)	-	
Urinary incontinence	1.6 (0.89)	-	

Data presented as mean (standard deviation). iNPH, Idiopathic normal pressure hydrocephalus; MMSE, Mini-mental state examination; mRS, modified Rankin Scale; iNPHGS, Idiopathic normal pressure hydrocephalus grading scale.

### 3.2. R2* differences across groups

**Figure 1:**
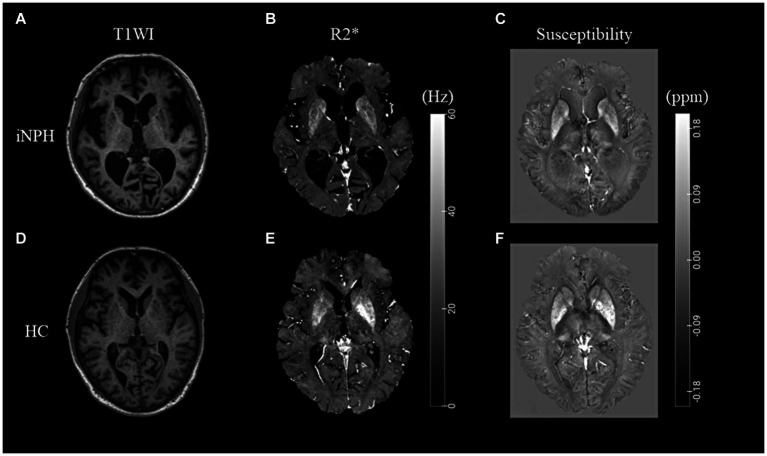
Representative T1-weighted images and R2* and susceptibility maps. Representative T1WI images **(A,D)**, R2* map **(B,E)**, and susceptibility map **(C,F)** for the iNPH and HC groups. iNPH, idiopathic normal-pressure hydrocephalus; HC, healthy control; T1WI, T1-weighted image.

**Figure 2:**
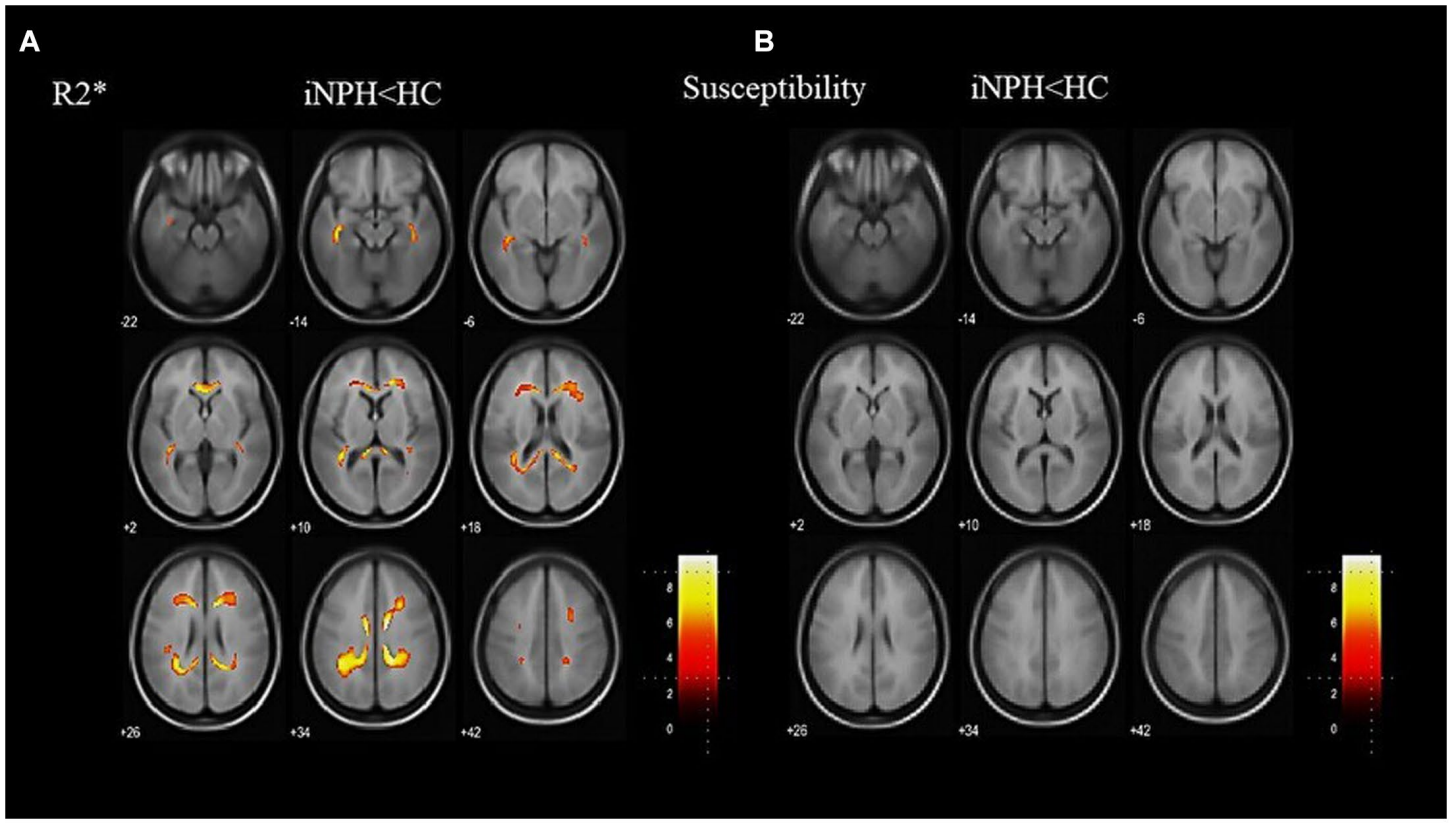
Results of a whole-brain group comparison of R2*. R2* is lower in the iNPH group than the HC group **(A)**. The specific anatomical regions are listed in Table 2. There are no significant differences in susceptibility values between the iNPH and the HC groups **(B)**. iNPH, idiopathic normal-pressure hydrocephalus; HC, healthy control.

Representative images of the T1-weighted images, R2* maps, and susceptibility maps for the iNPH and HC groups are shown in Figure 1. The voxel-based R2* comparisons between the groups yielded R2* values that were significantly lower in several WM regions, including the bilateral superior longitudinal fasciculus (SLF) and sagittal stratum (SS), in the iNPH compared to the HC group (FWE-corrected *p* < 0.05; Figure 2). The significant regions in the voxel-based comparisons between groups are listed in Table 2.

**Table 2:** Whole-brain comparisons of R2* between patients with iNPH and HC.

		Peak MNI coordinates				
Group comparison	Cluster size (number of voxels)	X	Y	Z	Peak T-value	Anatomical region
HC > iNPH	10,786	12	−16	36	9.81	Genu of corpus callosum
						Body of corpus callosum
						Splenium of corpus callosum
						Retrolenticular part of internal capsule, L
						Anterior corona radiata, R, L
						Superior corona radiata, R, L
						Posterior corona radiata, R, L
						Posterior thalamic radiation, R, L
						Sagittal stratum, L
						Cingulum, R, L
						Fornix/ Stria terminalis, L
						Superior longitudinal fasciculus, R, L
	452	39	−18	−15	6.48	Retrolenticular part of internal capsule, R
						Posterior thalamic radiation, R
						Sagittal stratum, R
						Fornix/Stria terminalis, R

iNPH, idiopathic normal pressure hydrocephalus; HC, healthy control; R, right; L, left.

### 3.3. QSM differences across groups

The voxel-based QSM comparisons between the groups showed no significant differences in susceptibility values (Figure 2). Before the atlas-based analysis, higher paramagnetic and local magnetic distortions caused by cerebral microbleeds (QSM > 1.0 ppm) were excluded. The results for WM susceptibility were either negative or near zero. The average susceptibility values for each of the JHU-WM atlas labels are listed in Supplementary File 1. These findings revealed no paramagnetic iron deposition in the WM of any subject.

### 3.4. Correlation between R2* and clinical presentations in patients with iNPH

**Figure 3:**
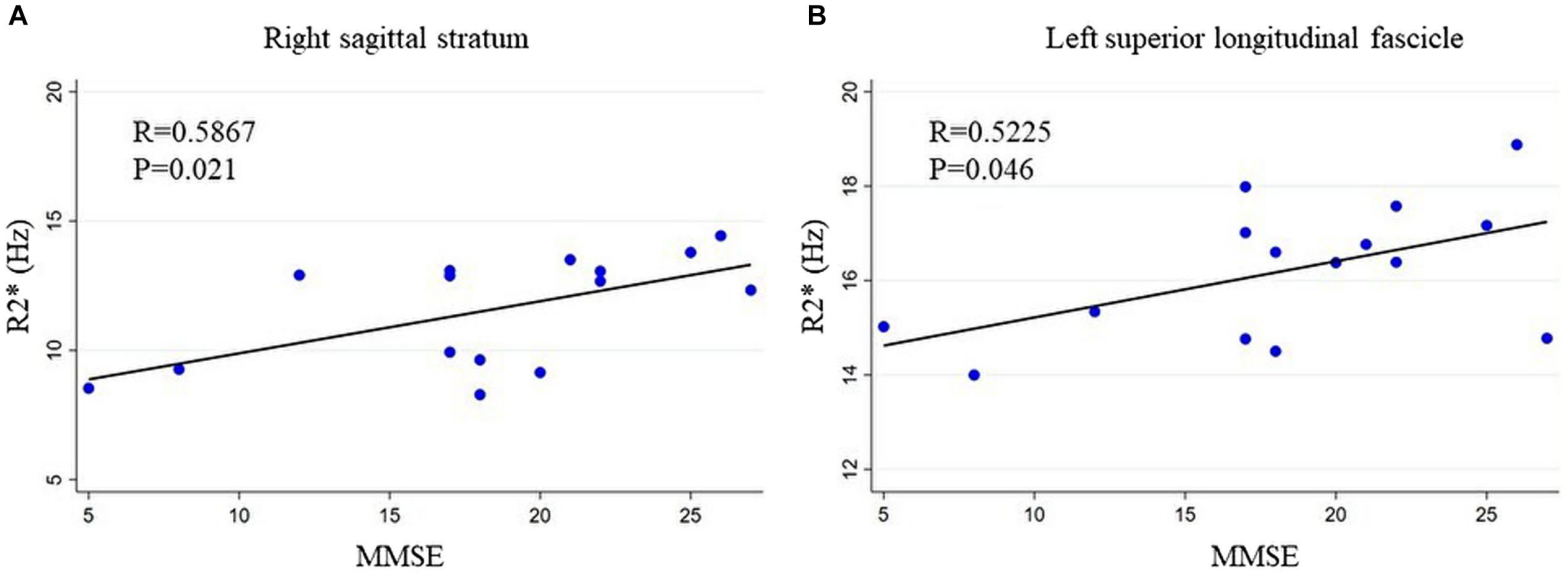
Relationship between MMSE and R2*. MMSE scores show a positive correlation with R2*. The relationship between R2* and MMSE scores in the **(A)** right sagittal stratum and **(B)** left superior longitudinal fascicle is shown. MMSE, mini-mental state examination.

Positive correlations between MMSE scores and R2* were observed in several WM regions, notably the left SLF and right SS, and MMSE scores had a positive correlation among patients with iNPH (*R* = 0.523, *p* < 0.05 in the left SLF and *R* = 0.587, *p* < 0.02 in the right SS; Figure 3), according to partial correlation analyses with covariate adjustment. Similarly, there was a negative correlation between INPHGS gait disturbance score and R2* in the left SS (*R* = −0.540, *p* < 0.05). There was no correlation between INPHGS urinary dysfunction score and R2*.

## 4. Discussion

In this study, voxel-based R2* values were compared between patients with iNPH and HCs to evaluate WM alterations. We found that R2* values in the iNPH group were lower in some WM regions, including the SLF and SS, and that microstructural changes without iron deposition in the SLF and SS were associated with cognitive impairment and gait disturbance in patients with iNPH.

According to the concepts of R2* relaxometry analysis, iron deposition as well as microstructural changes in WM, such as demyelination, are affected (9). In the voxel-based QSM comparisons, we found no significant differences in susceptibility values between the two groups. Additionally, the negative average susceptibility values in each group indicated that R2* depended exclusively on myelin content and accompanying microstructural changes. Furthermore, these findings suggests that myelin content and microstructural differentiation, rather than iron deposition, determine the susceptibility contrast in the WM in iNPH.

The significant decrease in R2* we observed in patients with iNPH in the voxel-based analysis indicates slight demyelination and extracellular space enlargement (10). Pathologically, damage to both the myelin and myelin-sheathed axons has been detected in iNPH (32, 33). Additionally, extracellular space enlargement can be described radiologically by increased MD in iNPH (5). Our findings are consistent with previous diffusion-based analyses of iNPH. Although water molecular diffusion can be used in DTI analyses to detect WM changes, quantitative DTI measures tend to render indispensable effects from a variety of factors (7). In contrast, R2* relaxometry analysis combined with QSM aims to separate microstructural changes from iron deposition, offering a more accurate and biologically true assessment of WM. However, the pathogenic significance of WM changes identified by R2* remains undetermined, and the exact cause of iNPH is still debatable. Chronic ventriculomegaly develops due to abnormal CSF dynamics, such as increased CSF pulsatility and decreased CSF drainage. One consequence is CSF diapedesis, which causes periventricular edema, disturbs normal brain homeostasis, has mass effects, and leads to local hypoperfusion/hypoxia. This crucial pathology leads to a series of sequential brain damage events, such as blood–brain barrier disruption, astrogliosis, neuroinflammation, and metabolic disturbance. All these events can cause white and gray matter lesions to form, which are the foundation for the clinical symptoms of iNPH (34).

This study observed an association between R2* changes and cognitive function in the SLF and SS. On diffusion MRI, the SLF has been linked to executive functions, which is also crucial to language and language disorders as well as the neurological basis of higher brain function in general (35, 36). On the other hand, SS is related to information processing speed (37) and may influence performance on the Frontal Assessment Battery (FAB) and the Trail Making Test (TMT) (35). Additionally, SS has been associated with nonverbal semantic processing, visuospatial processing, face recognition, and visual memory (38). Thus, our findings are consistent with previous studies that used DTI to assess cognitive dysfunction in patients with iNPH (39). This study also observed an association between R2* changes and gait disturbance in the SS. In a single photon emission computed tomography study, the supplementary motor area, medial primary sensorimotor area, the striatum, the cerebellar vermis, and the visual cortex were found to be activated during voluntary walking in normal subjects (40). Our findings suggest that SS-associated visuospatial cognitive function may be related to gait disturbance in iNPH. Previous DTI studies have reported that anterior thalamic radiation, forceps minor, anterior limb of the left internal capsule, left supplementary motor area, and corpus callosum regions were correlated with gait disturbance (41–43).

However, this study has several limitations. First, because we compared patients based on their clinical diagnosis, we could not directly confirm the correlation between clinical presentations and pathological changes. Future studies should attempt to relate pathological changes to the regions detected in this study (44). Second, we were unable to completely rule out the possibility of other causes of dementia in our participant group. iNPH can be complicated by vascular dementia or Alzheimer’s disease, but no criteria are available to ensure that these conditions are excluded. Third, we used MMSE scores as a proxy of cognitive dysfunction (45). However, the FAB has been reported to be sensitive to frontal lobe dysfunction and is already routinely used in reports, because executive dysfunction is a characteristic of cognitive dysfunction in patients with iNPH. Further evaluations are therefore expected to use tools such as the FAB and TMT instead. Fourth, we used INPHGS as an index of gait disturbance. For a more detailed evaluation, it is necessary to examine the relationship with the timed up and go test, as has been previously reported. Fifth, we did not evaluate therapeutic effects; the usefulness of this marker in indications for treatment needs to be examined in future studies. Further studies are also expected to evaluate the efficacy of shunting in patient groups similar to those we investigated here.

## 5. Conclusion

Our voxel-based group comparisons yielded a lower R2* in our iNPH group in some WM regions, including the SLF and SS, and showed that microstructural changes without iron deposition in these regions were associated with cognitive impairments. These findings indicate the possibility of using R2* relaxometry analysis for assessing cognitive impairment in iNPH. Further studies are required to establish the utility of R2* relaxometry as a biomarker of cognitive impairment in patients with iNPH.

## Data availability statement

The raw data supporting the conclusions of this article will be made available by the authors, without undue reservation.

## Ethics statement

The studies involving humans were approved by Institutional Review Board of Toyokawa City Hospital. The studies were conducted in accordance with the local legislation and institutional requirements. Written informed consent for participation was not required from the participants or the participants’ legal guardians/next of kin in accordance with the national legislation and institutional requirements.

## Author contributions

YK, YU, HK, KSa, and NM: study design. YK, SK, KSe, KM, TU, and KT: study conduction. YK, YU, HK, KSe, and SK: data analysis. YK, YU, HK, and NM: manuscript writing. All authors contributed to the article and approved the submitted version.

## Funding

This work was supported by the Japan Society for the Promotion of Science (JSPS) KAKENHI Grant Number 21K20908 (YK). The sponsor had no role in the study design; in the collection, analysis, and interpretation of data; in the writing of the report; and in the decision to submit the article for publication.

## Conflict of interest

The authors declare that the research was conducted in the absence of any commercial or financial relationships that could be construed as a potential conflict of interest.

## Publisher’s note

All claims expressed in this article are solely those of the authors and do not necessarily represent those of their affiliated organizations, or those of the publisher, the editors and the reviewers. Any product that may be evaluated in this article, or claim that may be made by its manufacturer, is not guaranteed or endorsed by the publisher.
