# Supplementary material for: Assessing white matter microstructural changes in idiopathic normal pressure hydrocephalus using voxel-based R2* relaxometry analysis
Source: Front Neurol. 2023 Sep 5;14:1251230. doi: 10.3389/fneur.2023.1251230 (PMC10507687; doi:10.3389/fneur.2023.1251230)
Supplement: Supplementary file 1 [file Table_1.DOCX]

**Supplementary file 1**

Mean susceptibility values and its standard deviations in the iNPH and HC groups obtained using the human white matter atlas created by Johns Hopkins University

| JHU  atlas labels | Structure | Susceptibility (ppb)  iNPH HC | |
| --- | --- | --- | --- |
| #1 | Middle cerebellar peduncle | -10.5(3.4) | -9.4(-4.1) |
| #2 | Pontine crossing tract | -12.9(8.9) | -24.5(8.2) |
| #3 | Genu of corpus callosum | -15.2(1.8) | -15.6(2.8) |
| #4 | Body of corpus callosum | -12.4(5.2) | -12.5(2.3) |
| #5 | Splenium of corpus callosum | -11.6(4.4) | -10.4(2.5) |
| #6 | Fornix | -18.6(12.6) | -19.6(7.5) |
| #7 | Corticospinal tract R | -12(7.6) | -21.8(6.6) |
| #8 | Corticospinal tract L | -13.2(8.9) | -22.8(6.9) |
| #9 | Medial lemniscus R | -10.1(5.9) | -16.3(6.9) |
| #10 | Medial lemniscus L | -11.2(5.8) | -17.3(7.1) |
| #11 | Inferior cerebellar peduncle R | -11.9(7.3) | -13.9(4.7) |
| #12 | Inferior cerebellar peduncle L | -12.6(6.4) | -13.6(6) |
| #13 | Superior cerebellar peduncle R | -24.5(11.2) | -26.7(5.3) |
| #14 | Superior cerebellar peduncle L | -26.1(10.6) | -27(4.7) |
| #15 | Cerebral peduncle R | -12.8(3.9) | -18.4(3.9) |
| #16 | Cerebral peduncle L | -14.9(5.8) | -18.1(5.1) |
| #17 | Anterior limb of internal capsule R | -10.3(3.2) | -12.4(3.6) |
| #18 | Anterior limb of internal capsule L | -10.8(3.2) | -11.5(3.6) |
| #19 | Posterior limb of internal capsule R | -16.5(4.5) | -19.9(8.1) |
| #20 | Posterior limb of internal capsule L | -14.4(7.8) | -18.7(6) |
| #21 | Retrolenticular part of internal capsule R | -15.7(5) | -11.6(4.8) |
| #22 | Retrolenticular part of internal capsule L | -13.5(4.5) | -9.6(4.1) |
| #23 | Anterior corona radiata R | -15.6(4.6) | -19.5(5) |
| #24 | Anterior corona radiata L | -17(5.2) | -20.3(5.1) |
| #25 | Superior corona radiata R | -20.8(5.9) | -15.4(3.2) |
| #26 | Superior corona radiata L | -20(5.5) | -15.2(2.8) |
| #27 | Posterior corona radiata R | -15.4(5.1) | -19.7(3.4) |
| #28 | Posterior corona radiata L | -17(4.1) | -20.6(4.2) |
| #29 | Posterior thalamic radiation R | -10.5(7.5) | -12.3(4.2) |
| #30 | Posterior thalamic radiation L | -9.5(6) | -8.9(2.9) |
| #31 | Sagittal stratum R | -17.3(4.5) | -21.1(5.7) |
| #32 | Sagittal stratum L | -14.5(4) | -16.3(3.1) |
| #33 | External capsule R | -12.5(3.3) | -13(3.6) |
| #34 | External capsule L | -10.5(2.6) | -11.2(3.9) |
| #35 | Cingulum (cingulate gyrus) R | -17.3(8.7) | -21.1(5.1) |
| #36 | Cingulum (cingulate gyrus) L | -17.4(7.7) | -22.5(4.8) |
| #37 | Cingulum (hippocampus) R | -16(6.1) | -14.5(6.1) |
| #38 | Cingulum (hippocampus) L | -17.7(6.7) | -14.7(4.1) |
| #39 | Fornix (cres) / Stria terminalis R | -17.2(6.6) | -19.8(7.7) |
| #40 | Fornix (cres) / Stria terminalis L | -14.6(6.7) | -19.8(7.1) |
| #41 | Superior longitudinal fasciculus R | -7.4(1.8) | -12.7(3.6) |
| #42 | Superior longitudinal fasciculus L | -8.1(2.3) | -11.1(3) |
| #43 | Superior fronto-occipital fasciculusR | -2.1(2.6) | -2.6(3) |
| #44 | Superior fronto-occipital fasciculus L | -2.6(2.9) | -3.2(3.7) |
| #45 | Uncinate fasciculus R | -7.8(7.4) | -16.5(9.5) |
| #46 | Uncinate fasciculus L | -7.4(7.1) | -17.9(9) |
| #47 | Tapetum R | -13.9(7.6) | -7.4(3.9) |
| #48 | Tapetum L | -14.5(6.8) | -7.2(4.3) |

JHU, Johns Hopkins University; iNPH, idiopathic normal pressure hydrocephalus; HC, healthy control
